# Predicting High Blood Pressure Using DNA Methylome-Based Machine Learning Models

**DOI:** 10.3390/biomedicines10061406

**Published:** 2022-06-14

**Authors:** Thi Mai Nguyen, Hoang Long Le, Kyu-Baek Hwang, Yun-Chul Hong, Jin Hee Kim

**Affiliations:** 1 Department of Integrative Bioscience & Biotechnology, Sejong University, 209 Neungdong-ro, Gwangjin-gu, Seoul 05006, Korea; mainguyen@sju.ac.kr; 2 Department of Computer Science & Engineering, Sejong University, 209 Neungdong-ro, Gwangjin-gu, Seoul 05006, Korea; lehoanglong@sju.ac.kr; 3 School of Computer Science & Engineering, Soongsil University, 369 Sangdo-ro, Dongjak-gu, Seoul 06978, Korea; kbhwang@ssu.ac.kr; 4 Department of Preventive Medicine, College of Medicine, Seoul National University, Seoul 03080, Korea; ychong1@snu.ac.kr; 5 Institute of Environmental Medicine, Seoul National University Medical Research Center, Seoul 03080, Korea

**Keywords:** DNA methylome, machine learning, deep learning, high blood pressure

## Abstract

DNA methylation modification plays a vital role in the pathophysiology of high blood pressure (BP). Herein, we applied three machine learning (ML) algorithms including deep learning (DL), support vector machine, and random forest for detecting high BP using DNA methylome data. Peripheral blood samples of 50 elderly individuals were collected three times at three visits for DNA methylome profiling. Participants who had a history of hypertension and/or current high BP measure were considered to have high BP. The whole dataset was randomly divided to conduct a nested five-group cross-validation for prediction performance. Data in each outer training set were independently normalized using a min–max scaler, reduced dimensionality using principal component analysis, then fed into three predictive algorithms. Of the three ML algorithms, DL achieved the best performance (AUPRC = 0.65, AUROC = 0.73, accuracy = 0.69, and F1-score = 0.73). To confirm the reliability of using DNA methylome as a biomarker for high BP, we constructed mixed-effects models and found that 61,694 methylation sites located in 15,523 intragenic regions and 16,754 intergenic regions were significantly associated with BP measures. Our proposed models pioneered the methodology of applying ML and DNA methylome data for early detection of high BP in clinical practices.

## 1. Introduction

High blood pressure (BP) is a leading risk factor for morbidity and mortality worldwide, causing a major public health challenge that demands interdisciplinary efforts [1]. It has been predicted that the global burden of hypertension will be around 1.6 billion by 2025, accounting for 29% of the world’s population [2]. According to a recent analysis of trends in hypertension prevalence, the number of adults aged from 30 to 79 years old with hypertension has doubled to nearly 1.3 billion in the last 30 years [3]. However, only 46.5% of people with high BP are aware of this problem until it has reached a dangerous level, because there are no warning signs or symptoms [4]. Prior work has estimated that less than 1 in 5 persons with hypertension is being monitored [1]. Existing evidence has indicated that early detection of high BP is an effective strategy for preventing and managing hypertension [5].

Clinically diagnosing suspected subjects as hypertension patients has been mostly performed at clinics using either a validated oscillometric upper-arm cuff or a calibrated auscultatory device, because these methods are convenient and the instruments used are cheap. In addition, they can be easily applied on a large scale. However, white-coat hypertension (i.e., elevated office BP but normal out-of-office BP) and masked hypertension (i.e., normal office BP but elevated out-of-office BP) could not be detected using these kinds of diagnosis methods. For such cases, applications of 24 h ambulatory BP and home BP monitoring are required to confirm the diagnosis clinically [4]. Wearing the device continuously for 24 h may cause discomfort and soreness to patients as well as a potential systemic bias related to a loose arm cuff at specific timepoints. Therefore, novel approaches of using biomarkers to assist early detection of high BP have received great attention in the past decade.

It is widely known that epigenetic modifications play an important role in the biological pathway of hypertension [6]. Among these modifications, DNA methylation is the best studied [7]. The DNA methylation process involves adding a methyl group to the cytosine base at a region including repeated cytosine-guanine bonds (CpG island). When a gene is heavily methylated, it tends to remain in a transcriptionally silent state. In response to environmental factors, methylation level can change dramatically [8]. Promoter CpG island (i.e., clusters of CpG sites located in the promoter) methylation is considered a potential type of biomarkers for disease detection, subtype classification, prognosis, and treatment response prediction [9].

Recent studies have discovered significant associations between DNA methylations and BP variations [7,8,10,11,12,13]. Han et al. [7] highlighted an important role of gene-specific DNA methylation in the pathogenesis of high BP in relation to angiotensin-converting enzyme [14], lipid and amino acid metabolism [15], and dysfunction of glucose metabolism [16]. A subsequent review has concluded that DNA methylation is an epigenetic mediator in the pathogenesis of systemic hypertension [8]. Richard et al. [10] identified 13 replicated CpG sites that could explain 1.4% and 2.0% of interindividual variations in systolic BP (SBP) and diastolic BP (DBP), respectively. More interestingly, emerging evidence has indicated that DNA methylation is significantly associated with lifestyle habits (e.g., smoking, drinking, and diet), aging, obesity, and sex, all of which are important risk factors for high BP [7]. Kim et al. [17] found the association between DNA methylation in peripheral blood leukocytes and prevalence of hypertension, indicating a potential of DNA methylation as a biomarker for high BP.

Machine learning (ML) has emerged to play a vital role in bioinformatics due to its ability to handle exponentially increasing amount of data [18]. Many researchers have applied ML to DNA data [19,20,21,22]. In particular, several applications of ML in epigenomics have assisted medical professionals and researchers to perform human disease-related tasks such as disease detection, subtype classification, prognosis, and treatment response prediction [23,24,25,26,27,28]. Given the success of existing ML models for detecting breast cancer [29,30], lung cancer [31], coarctation [32], concussion [33], and schizophrenia [34], we proposed DNA methylome-based predictive models using three common ML algorithms including deep learning (DL), random forest (RF), and support vector machine (SVM) for high BP prediction in this study.

## 2. Materials and Methods

### 2.1. Study Participants

DNA methylome and BP data were obtained from 50 elderly individuals approved for this study among 60 elderly individuals who participated in a previous randomized crossover trial study [35]. They were all at least 60 years old without having any medical history for heart diseases, cancer, liver diseases, and endocrine diseases.

Details about our study design were described in a previous publication [35]. In brief, participants were asked to visit the study site three times, at one-week intervals. At each visit, their blood samples were collected and their BPs were measured twice after they stayed in a sedentary position for ≥ 10 min using an automatic sphygmomanometer (HEM-780: Omron, Kyoto, Japan) with a standard cuff. If the two measurements differed by ≥ 5 mmHg, a third measurement was performed to ensure the reliability of results. Means of measurements were used for analysis. Participants were considered to have high BP if they met at least one of the following criteria: (1) a history of hypertension diagnosed, (2) SBP ≥ 140 mmHg, and (3) DBP ≥ 90 mmHg.

### 2.2. DNA Methylome Level Measurements

A total of 150 blood samples were stored for DNA methylome profiling using an Illumina Infinium HumanMethylation450 BeadChip in accordance with the manufacturer’s protocol (Illumina Inc., San Diego, CA, USA). In brief, the quality of each DNA sample was initially checked using a NanoDrop^®^ ND-1000 UV-Vis Spectrophotometer. Qualified DNA samples (500 ng) were then bisulfite-converted using a Zymo EZ DNA methylation kit. They were then amplified and hybridized for BeadChips. Subsequently, fluorescently stained BeadChips were scanned by Illumina iScan scanner following standard Illumina procedures (iScanTM System, https://sapac.illumina.com/systems/ array-scanners/iscan.html). Image intensities were extracted using Illumina’s GenomeStudio software version 2011.1 (methylation model version 1.9.0) (Illumina Korea, Seoul, Korea). Eventually, the methylation level of each CpG site was calculated as a ratio of methylation intensity to total methylation and unmethylation intensities.

Because of varying characteristics between two types of assays simultaneously used in the Infinium HumanMethylation450 array, a sequence of cleaning methods for DNA methylation data were applied according to the manufacturer’s suggestions to reduce a systemic bias, as follows (Infinium^®^ HumanMethylation450 BeadChip, Illumina, Inc., San Diego, CA, USA):

Within-array normalization: Raw data were background-corrected and then dye-bias equalized using Methylumi [36] and Lumi [37] packages in R, respectively.

Filtering: To reduce threats of artifactual data, CpG sites with detection *p*-values (hereafter *p*-values) ≥ 0.05 displaying in at least 25% of all samples were filtered out [38]. In further details, the *p*-value was generated for every CpG site in every sample to evaluate the magnitude of the signal by comparing the total signal level (i.e., sum of unmethylated intensity and methylated fluorescent intensity) with the background signal level, which was estimated using negative control probes. A smaller *p*-value indicated a more reliable signal, while a high *p*-value indicated a low-quality signal. Additionally, CpG sites with missing values in at least one sample and CpG sites on X or Y chromosome were excluded to ensure a strict dataset and to avoid a potential gender-specific bias of the dataset, respectively.

Between-array normalization: Filtered data were normalized with beta-mixture quantile dilation method that could adjust β-values of type II assay into a statistical distribution characteristic of type I assay using a three-state beta-mixture model [39].

### 2.3. Nested Cross-Validation

Because of a small sample size, we applied a nested cross-validation (CV) to estimate an unbiased generalization performance. The dataset was initially divided into five groups, with roughly the same ratio (1.4:1) of those with high BP to those without high BP using a random split. To avoid biological bias due to a participant being sampled three times, data obtained from the same participant were allocated in the same group. In other words, each group consisted of 10 participants with 30 samples.

There was a total of five outer CVs. In an outer CV, when a group was used as a test set for estimating prediction results (i.e., outer test set), the four remaining groups (i.e., outer training set), which constructed an inner loop CV, were used for hyperparameter tuning. Of these four groups, one was subsequently used as inner test set and the remaining three groups were used as inner training set. The optimal model for each outer CV was selected based on its performance on the inner test set.

### 2.4. DNA Methylome Data Preprocessing

To avoid data leakage, we performed preprocessing using data obtained from 120 samples in the outer training sets of each outer CV separately. After normalizing DNA methylome data using a min–max scaler, we reduced the dimensionality of the data using principal component analysis (PCA), a simple unsupervised method that could find a low-dimensional representation (i.e., principal components) of the input data on condition that as much of the information as possible was captured [40].

### 2.5. Predictive Models

Three common supervised ML algorithms including DL, RF, and SVM were developed for high BP prediction in Python 3.9 using PyTorch, Numpy, Scikit-learn, and Pandas. The code of our study is publicly available in Github (https://github.com/lehoanglong95/high_blood_pressure_prediction, accessed on 27 February 2022).

Among DL architectures, a multi-layer perceptron (MLP) composed of an input layer, multiple hidden layers, and an output layer was proposed. Specifically, input nodes represented CpG sites that were significantly associated with high SBP and/or high DBP, whereas a binary variable in the output layer indicated the presence or the absence of a high BP. In the hidden and output layers, two common activation functions, the rectified linear unit (ReLU) [41] and the sigmoid function [42], were employed in the same order. The ReLU was used to convert all negative values to zero with the positive values remaining between layers, whereas the sigmoid function was used to generate values from 0 to 1. A threshold of 0.5 was then applied to obtain binary values. We utilized a binary cross-entropy loss function and Adam optimizer, an algorithm for stochastic optimization known to be straightforward to implement, which is computationally efficient, and which requires little memory and little tunning of hyperparameters to optimize the model parameters [43]. The initial learning rate was $\alpha =1\times 10^{-3}$. Other hyperparameters were β1 = 0.9, β2 = 0.999, and ε = $1\times 10^{-8}$. To optimize the learning process, the learning rate was reduced by 10 times every 75 epochs. Batch size was set to be 5. Batch normalization was also utilized in the proposed DL models. After training procedure, optimal DL models achieving the lowest loss on the inner test sets were evaluated using the outer test sets.

RF is a supervised learning algorithm that can randomly create a great number of relatively uncorrelated decision trees at the training stage [40]. In a classification task, as each tree generates a class prediction, RF selects the class with the highest number of votes as the final prediction result. Our proposed RF was constructed with 100 trees. Each tree had the maximum depth of 100. As for SVM, we used linear kernel to construct hyperplanes in a multidimensional space, which allowed us to classify the output into having or not having a high BP [44].

### 2.6. Model Evaluation

Due to the small sample size, performances of the proposed models were assessed using the area under the receiver operator characteristics curve (AUROC), the area under the precision-recall curve (AUPRC), accuracy (i.e., evaluated overall classification performance), and F1-score Micro Average calculated by counting sums of True Positives, False Negatives, and False Positives across all classes (i.e., weighted the sensitivity and precision of the model evenly) [45].

For each predictive model, we calculated means and standard deviations for each evaluation metric using performance with five outer test sets. To make straight comparisons between predictive results of three models, we applied a multiple paired *t*-test using R version 4.1.2 (https://www.r-project.org/, accessed on 27 February 2022) with significance level set at *p*-value < 0.05.

### 2.7. Functional Analyses

To confirm the reliability of using DNA methylome as biomarkers for high BP, we constructed two mixed-effects models and evaluated the associations between DNA methylation level and SBP and/or DBP measures. In both models, potential confounding factors were selected in an a priori manner. Finally, age, sex, body mass index, sequence of visit, visit date, a history of hypertension, and a history of diabetes were adjusted. SAS version 9.4 (SAS Institute Inc., Cary, NC, USA) was used for statistical analyses with significance level set at *p*-value < 0.05.

CpG sites that showed significant associations with SBP and/or DBP were mapped with target genes using the manufacturer’s database (Illumina, Inc., San Diego, CA, USA). Next, the gene list was uploaded to DAVID Bioinformatics Resources 6.8 (https://david-d.ncifcrf.gov/, accessed on 27 February 2022) to discover disease classes regulated by target genes. Afterwards, we constructed a functional network to visualize associations between genes and high BP-related diseases using DisGeNet version 7.0 (Integrative Biomedical Informatics Group, Barcelona, Spain) (https://www.disgenet.org/, accessed on 27 February 2022) and Cytoscape version 3.9.0 (U.S. National Institute of General Medical Sciences, Bethesda, MD) (https://cytoscape.org/, accessed on 27 February 2022).

## 3. Results

### 3.1. Study Participants

Main characteristics and BP measurements of 50 participants are presented in Table 1. The majority (n = 47, 94%) of participants were non-smoking females. Those who were overweight and/or obese (body mass index ≥ 25 kg/m2) accounted for 50%. Twenty-six (52%) and forty-two (84%) participants had a history of hypertension and diabetes, respectively. Among 150 BP measures obtained from 50 participants, 48 (32%) and 31 (20.7%) SBP measures were elevated (130–139 mmHg) and high (≥140 mmHg), respectively, while 125 (83.3%) DBP measures were normal (<85 mmHg). According to classification criteria described above, 58% of BP measures were considered high BP.

### 3.2. Predictive Models for High BP Detection

**Figure 1:**
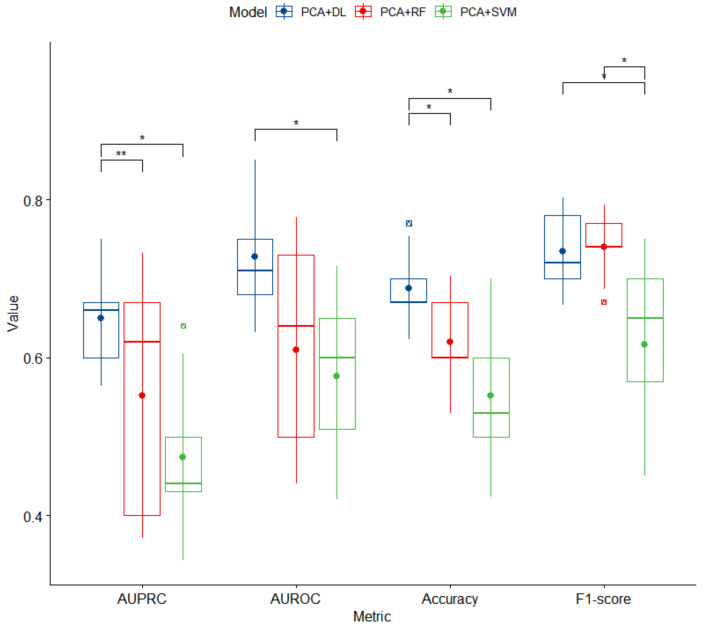
Box plots of performance of three proposed models for high BP prediction. PCA, principal component analysis; DL, deep learning; RF, random forest; SVM, support vector machine; AUPRC, area under the precision-recall curve; AUROC, area under the receiver operator characteristics curve. An asterisk (*) denotes a significant difference with *p*-value < 0.05. A double asterisk (**) denotes a significant difference with *p*-value < 0.005.

Hyperparameters of optimal DL models selected based on their performances with the inner test sets are presented in Table S1. Performances of three proposed models with the outer test sets are summarized in Figure 1. Overall, DL significantly outperformed RF and SVM based on AUPRC and accuracy (mean ± SD) (for DL, AUPRC = 0.65 ± 0.06 and accuracy = 0.69 ± 0.05; for RF, AUPRC = 0.55 ± 0.13 and accuracy = 0.62 ± 0.06; for SVM, AUPRC = 0.47 ± 0.09 and accuracy = 0.55 ± 0.09; *p* < 0.05). There were significant differences in AUROC and F1-score Micro Average between DL and SVM (mean ± SD) (for DL, AUROC = 0.73 ± 0.07 and F1-score = 0.73 ± 0.05; for SVM, AUROC = 0.58 ± 0.10 and F1-score = 0.62 ± 0.10; *p* < 0.05).

### 3.3. CpG Sites Significantly Associated with High BP

There were 37,610 and 40,530 CpG sites significantly associated with SBP and DBP, respectively. Of these, 16,446 CpG sites were significantly associated with both SBP and DBP as presented in the Venn Diagram (Figure S1). We identified DNA methylation at 61,694 CpG sites showing significant associations with SBP and/or DBP located in 15,523 intragenic regions and 16,754 intergenic regions.

**Figure 2:**
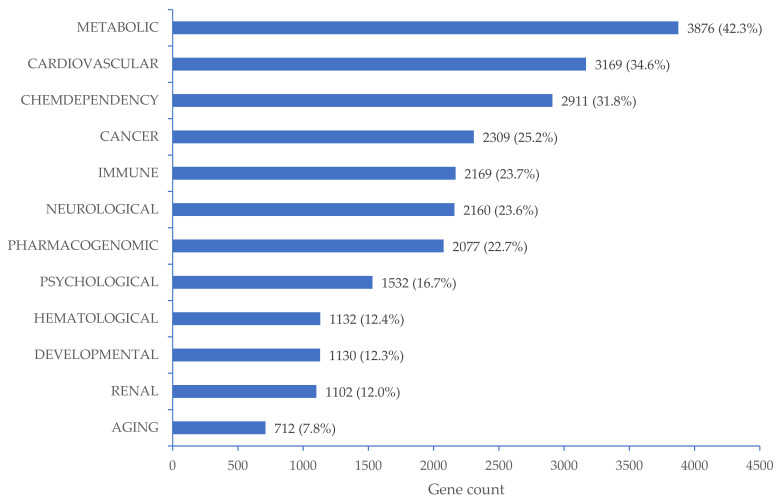
Disease classes related to 9154 target genes mapped with CpG sites significantly associated with high BP. The percentages were calculated as the number of target genes that regulated the corresponding disease class divided by 9154 target genes that regulated all related disease classes.

Among 15,523 genes, 9154 were found to be significantly related to 12 disease classes in DAVID Bioinformatics Resources 6.8 database (Figure 2), including 3169 (34.6%) genes involved in the regulation of cardiovascular diseases (Table S2). We found that 564 genes were significantly related to hypertension (Table S2). Comprehensive gene ontology in terms of biological process, cellular component, and molecular function as well as KEGG pathway for a total of 15,523 target genes can be found in Figure S2.

**Figure 3:**
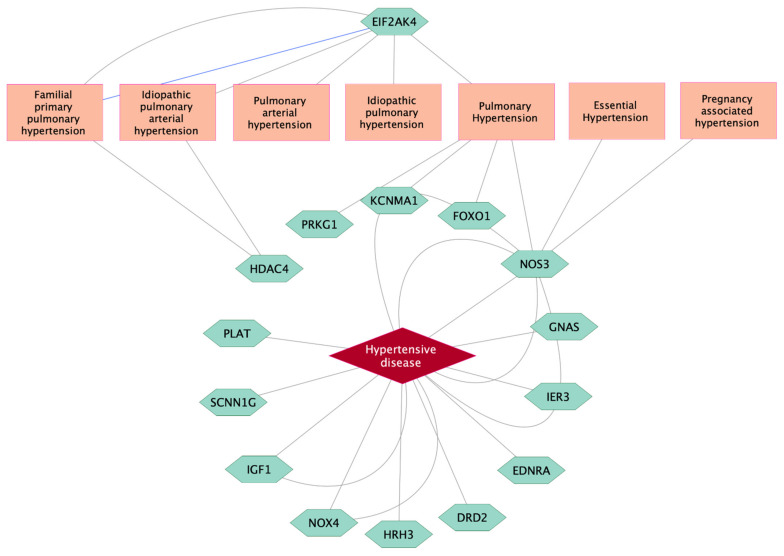
Target genes as significant biomarkers for high BP-related diseases using a curated database in the DisGeNet platform. Grey lines link gene expression biomarkers for target disease, while blue line links genetic variation for target disease.

Among 1037 genes showing significant relationships with high BP-related diseases in the DisGeNet database, 15 genes (*NOS3, GNAS, IER3, EDNRA, DRD2, HRH3, NOX4, IGF1, SCNN1G, PLAT, HDAC4, PRKG1, KCNMA1, FOXO1,* and *EIF2AK4)* were classified as significant biomarker genes for high BP-related diseases (Table S2). Detailed relationships between 15 biomarker genes and target diseases are visualized in Figure 3. There was strong evidence indicating that *NOS3* was a reliable biomarker for hypertensive diseases, followed by *IGF1, NOX4, GNAS, IER3, EDNRA, DRD2, HRH3, SCNN1G,* and *PLAT*. NOS3 was also considered a biomarker for pulmonary, essential, and pregnancy-associated hypertension. *EIF2AK4* significantly regulated the pulmonary hypertension-related disease class. Biomarker genes for pulmonary hypertension included *PRKG1, KCNMA1, FOXO1,* and *NOS3*. There were significant relationships between *HDAC4* and two specific types of pulmonary hypertension: familial primary pulmonary hypertension and idiopathic pulmonary arterial hypertension.

Table S3 shows detailed mapping information as well as estimation of magnitude of associations between significant CpG sites and BP measures. There were 140 significant CpG sites located in 15 biomarker genes for high BP-related diseases found in DisGeNet platform (Table S3). Because there might be multiple CpG sites located in a downstream target gene, for each biomarker gene, we selected a corresponding CpG site with the strongest association with BP (i.e., the biggest absolute value of the estimation) and/or significantly associated with both SBP and DBP measures with *p*-value < 0.05 to present in Table 2. Except for cg16655193 located in *IGF1*, which was negatively associated with only SBP along with a set of CpG sites that were positively associated with only DBP (cg03573792, cg18899064, and cg18248586, respectively located in *EDNRA, PLAT*, and *DRD2*), all remaining CpG sites were significantly associated with both SBP and DBP measures (*p* < 0.05). It was found that cg20203971 in *HDAC4* (for SBP, estimate = 443.9, *p* < 0.001; for DBP, estimate = 205.5, *p* < 0.001) and cg04956913 in *IER3* (for SBP, estimate = –413.3, *p* = 0.011; for DBP, estimate = –202.1, *p* = 0.023) showed the strongest positive and negative associations with BP, respectively.

## 4. Discussion

Given an increasing burden of hypertension worldwide [46], there have been great efforts made towards the early detection of the “silent killer” following advancements of ML. The performance of our proposed DNA methylome-based DL model was comparable to those of some existing predictive models for high BP using demographic, lifestyle, and biochemical data [47,48,49,50,51]. For example, compared with the latest MLP for hypertension prediction developed by López-Martínez et al. [51], which achieved an accuracy of 0.73, a recall of 0.40, a precision of 0.58, an F1-score of 0.47, and an AUROC of 0.77 using data obtained from the National Health and Nutrition Examination Survey from 2007 to 2016 with 24,434 participants, our proposed MLP was slightly better, with an accuracy of 0.69, a recall of 0.77, a precision of 0.72, an F1-score of 0.73, and an AUROC of 0.73. However, it could be inappropriate to make a direct comparison between the two models using different input data. Indeed, all existing models used demographic characteristics (e.g., age, gender, employment, education level), lifestyle (e.g., physical activity, tobacco use, alcohol use, dietary habit), and biochemical parameters (e.g., total cholesterol, lipoprotein, triglyceride levels) as input data [47,48,49,50,51], while our study was the first to take advantage of DNA methylation data as biomarkers for high BP detection. A significant upside of demographic, lifestyle, and biochemical data is that they are easier, more convenient, and cheaper to be collected, leading to very large datasets. By contrast, our dataset is quite small because it is expensive and complex to obtain DNA methylation levels. Limited and imbalanced data of 150 samples obtained from only 50 participants posed a challenge for developing ML models, especially DL. Although the contribution of the first type of input data to mechanism of development of hypertension remains unclear, DNA methylome data could be highly sensitive and biologically explained, making it widely considered as novel biomarkers for hypertension [17]. Furthermore, public databases of demographic and clinical data might have several limitations [49]. First, as such datasets often provide standardized data only, a shortage of raw data related to key demographic factors could limit numbers of valuable factors for training predictive models. Second, adjustments for patient data (e.g., performing up to three biochemical measures per patient for cross-checking) might increase input nodes, thus increasing processing time. Interestingly, recent studies have found that DNA methylome can be potentially used as biomarkers not only for hypertension, but also for a wide range of non-communicable diseases such as cancer [52] and type 2 diabetes [53]. Our predictive models pioneered a comprehensive approach for assisting clinical practices using only a blood sample to obtain DNA methylome data for multiple disease prediction at a time. Nevertheless, we adjusted for potential confounding factors selected in an a priori manner in the mixed-effects models to confirm the reliability of using DNA methylome as biomarkers for high BP and found 15,523 intragenic regions showing significant associations with SBP and/or DBP. Among 15,523 intragenic regions, 3169 regions involved in the regulation of cardiovascular diseases, with 564 significantly related to hypertension. However, the significant relationships with cardiovascular diseases including hypertension disappeared when those potential confounding factors were not adjusted (data not shown here). From the results, we could expect that the integration of those potential confounding factors in the model yielded better accuracy. Nevertheless, 0.73 in AUROC found in our study was still quite low in general, and thus in the future, we need to find additional confounding factors and integrate those to use as fully automated pipelines for the task at hand.

Among DNA methylome-based ML models for high BP prediction proposed in this study, we found that DL outperformed RF and SVM overall, consistent with results obtained from a previous study conducted by Ture et al. [50] who compared performances of four statistical algorithms, three decision trees, and two neural networks in terms of sensitivity, specificity, and predictive rate and concluded that MLP yielded better results than all other techniques. Despite its superior performance, the black-box nature of DL remains a question. Little has been known about the contribution of each CpG site to the prediction results, posing a lack of interpretability. In our study, the biggest limitation was the very small sample size, as it might limit learning ability of predictive models. Furthermore, we could not stratify our subjects according to sex and smoking status because of small sample size. If the sample size became larger, the variations in machine learning models could be smaller and it could lead to better test performance. Moreover, a larger sample size will uncover the functional and causal relationships between DNA methylation and BP [10]. Furthermore, because of limited statistical power due to the relatively small sample size in our study, our analyses results need to be reconfirmed through replication in another sample of Koreans [12]. In addition, less computationally demanding methods, for example, logistic regression, naive Bayes, and stochastic gradient descent, could sometimes outperform more vaunted tools in the dimensionality reduction step. Therefore, future research should be conducted considering stratification by several factors such as sex and smoking status, using various dimensionality reduction methods with a larger dataset. It is worth noting that thanks to the availability of cancer-related data provided by open-access databases such as The Cancer Genome Atlas (TCGA) [54] and the Gene Expression Omnibus (GEO) [55], a wide range of predictive models focusing on predicting cancer (e.g., breast cancer, lung cancer, liver hepatocellular carcinoma, and kidney clear cell carcinoma) using DNA methylome data have been successfully developed [29,30,31,56,57]. This indicates that the limited data issue can be addressed with an establishment of similar databases for hypertension.

Our findings from functional analyses can strengthen the application of DNA methylome as biomarkers for high BP. The list of CpG sites showing significant associations with BP in mixed-effects models covered existing DNA methylation biomarkers in hypertension [7,58]. Such DNA methylation sites primarily participated in regulating hypertension via three biological pathways related to the etiology of hypertension, including renin-angiotensin-aldosterone system (RAAS), renal sodium retention system, and sympathetic nervous system. While the first pathway is well known to be involved in hypertension occurrence [59], the other two pathways are mostly involved in hypertension pathogenesis and pathophysiology [60,61]. As a key enzyme in the RAAS, angiotensin-converting enzyme (ACE) plays an important role in BP regulation [59]. In line with results obtained from a previous study conducted by Rangel et al. [62], we found inverse associations of DNA methylation (cg19354750 and cg23524341) with *ACE* activity, SBP, and DBP. Hypomethylation of the angiotensinogen gene (AGT) promoter can activate *AGT* expression in adipose-induced hypertension [63]. Three CpG sites (cg07502417, cg01083716, and cg24474852) were discovered to be significantly associated with SBP in our analyses. Among three subunits composing adducin (ADD), a mutation of the α-subunit encoded by *ADD1* can lead to an increase in renal sodium reabsorption, subsequently causing hypertension [64]. *ADD1* also directly participates in the pathophysiology of hypertension [64]. Lower *ADD1* promoter DNA methylation has been found to be related to higher risk of essential hypertension [15]. Among CpG islands located in *ADD1* gene promoter, cg03889700 was found to be negatively associated with DBP in our analysis.

With regard to 15 biomarker genes for high BP-related diseases found in DisGeNet database, CpG sites located in *IER3* (cg00687252, cg27545367, and cg04956913) and *PRKG1* (cg15583492, cg05867154, cg11486694, and cg06976598) were all negatively associated with BP, while CpG sites located in *PLAT* (cg18899064) and *DRD2* (cg18248586) were positively associated with BP. Arlt and Schäfer [65] indicated that the ablation of *IER3* can induce changes in BP control and hypertension in mice. However, little is known about their association in humans. *PRKG1* plays a vital role in regulating the contractility of vascular smooth cell as well as nitric oxide signaling in cardiovascular homeostasis [66]. *PRKG1* deficiency can result in pulmonary hypertension via the activation of Rho A/Rho kinase signaling pathway [67]. For the remaining biomarker genes, there were both positive and negative CpG sites in relation to BP. Based on the magnitude of the estimation, the strongest positive association was found between cg20203971 located in *HDAC4* and SBP, while the strongest negative association was found between cg01995660 located in *FOXO1* and SBP. Both genes were found to be significantly associated with pulmonary-related hypertension in DisGeNet database. In further detail, Usui et al. [68] indicated that in spontaneous hypertensive rats, *HDAC4* can induce proinflammatory responses, which might mediate the development of hypertension. It has been found that *FOXO1* can control BP via its regulation of angiotensinogen and angiotensin II [69].

Compared with 13 CpG sites identified in a previous study for BP regulation [10], we found a consistent result for cg17061862 located in the intergenic region of chromosome 11, which was positively associated with both SBP and DBP in the present study. Differences in associations between 12 remaining CpG sites and BP could be attributable to characteristics of participants. Richard et al. [10] recruited 17,010 individuals of African American, European, and Hispanic ancestry, while our study participants were elderly Korean people. Although the robustness of our analyses was confirmed by consistent evidence in DAVID Bioinformatics Resources 6.8 and DisGeNet databases, we were only able to determine the existence of associations. Future studies with larger cohorts of Korean population are needed to confirm whether DNA methylation status at CpG sites discovered in the present study could affect BP measures.

## 5. Conclusions

This is the first study to propose ML-based approaches to take advantage of DNA methylation level to predict high BP. Our analyses discovered 61,694 methylation sites located in 15,523 intragenic regions and 16,754 intergenic regions significantly associated with BP measures and confirmed the reliability of using DNA methylome as a biomarker for high BP. Among three ML algorithms, DL achieved the highest performance with the test set, showing AUROC, AUPRC, accuracy, and F1-score of 0.73, 0.65, 0.69, and 0.73, respectively. These results were comparable to performances of existing predictive models for high BP using demographic, lifestyle, and biochemical data, suggesting the potential applicability of a DNA methylome-based DL model in clinical practices for hypertension management.

## Figures and Tables

**Table 1:** Information about study participants.

Variables	Parameter (n = 50)
Age, year, mean ± SD	72.5 ± 3.5
Sex, n (%)	
Male	3 (6.0)
Female	47 (94.0)
BMI, kg/m^2^, n (%)	
18.5–<23.0	11 (22.0)
23.0–<25.0	14 (28.0)
25.0–<30.0	22 (44.0)
30.0–<35.0	3 (6.0)
Drinker, n (%)	
Yes	12 (24.0)
No	38 (76.0)
Smoker, n (%)	
Yes	3 (6.0)
No	47 (94.0)
History of hypertension, n (%)	
Yes	26 (52.0)
No	24 (48.0)
History of diabetes, n (%)	
Yes	42 (84.0)
No	8 (16.0)
Current SBP, mmHg, mean ± SD	128.5 ± 66.5
<130, n (%)	71 (47.3)
130–139, n (%)	48 (32.0)
≥140, n (%)	31 (20.7)
Current DBP, mmHg, mean ± SD	77.3 ± 38.5
<85, n (%)	125 (83.3)
85–89, n (%)	16 (10.7)
≥90, n (%)	9 (6.0)
Current high BP status, n (%)	
Yes	87 (58.0)
No	63 (42.0)

SD, standard deviation; BMI, body mass index, BP, blood pressure; SBP, systolic blood pressure; DBP, diastolic blood pressure; SD, standard deviation. Current BP measure of a participant was considered high if it met at least one of the following criteria: (1) the participant had a history of hypertension diagnosed, (2) SBP ≥ 140 mmHg, and (3) DBP ≥ 90 mmHg.

**Table 2:** Estimated associations between BP measures and the most significant CpG sites mapped with biomarker genes for high BP.

CpG	Chr	Position	USCS Gene	SBP			DBP		
				Estimate	SE	*p*-Value	Estimate	SE	*p*-Value
cg20203971	2	240171099	*HDAC4*	443.9	101.0	<0.001	205.5	56.0	<0.001
cg03573792	4	148465429	*EDNRA*	99.1	57.7	0.088	64.4	31.3	0.041
cg04956913	6	30712436	*IER3*	–413.3	161.2	0.011	–202.1	88.1	0.023
cg13224213	7	150689881	*NOS3*	–86.3	41.7	0.040	–57.7	22.5	0.012
cg18899064	8	42066228	*PLAT*	58.2	70.4	0.410	91.3	37.6	0.016
cg07528661	10	78647708	*KCNMA1*	152.6	65.7	0.022	71.9	35.9	0.047
cg06976598	10	53639124	*PRKG1*	–37.3	13.8	0.008	–19.6	7.5	0.010
cg18248586	11	113329026	*DRD2*	64.4	33.4	0.056	47.2	18.0	0.010
cg03793270	11	89224684	*NOX4*	43.4	20.9	0.040	26.0	11.3	0.023
cg16655193	12	102802953	*IGF1*	–115.9	52.5	0.029	–52.5	28.7	0.070
cg07109046	13	41204388	*FOXO1*	50.8	15.1	0.001	23.1	8.3	0.006
cg10821964	15	40269214	*EIF2AK4*	177.0	70.2	0.013	102.4	38.1	0.008
cg09094674	16	23194733	*SCNN1G*	78.7	30.3	0.010	64.0	16.0	<0.001
cg20019489	20	57414351	*GNAS*	–47.0	16.6	0.005	–21.1	9.1	0.022
cg09640960	20	60794676	*HRH3*	162.0	48.5	0.001	80.9	26.6	0.003

USCS, University of California, Santa Cruz; SBP, systolic blood pressure; DBP, diastolic blood pressure; SE, standard error.

## Data Availability

The datasets analyzed during the current study are not publicly available due to protection of participant confidentiality but are available from the corresponding author on reasonable request with assurances and plans in place to protect confidentiality.

## Supplementary Materials

The following supporting information can be downloaded at: https://www.mdpi.com/article/10.3390/biomedicines10061406/s1, Figure S1: Numbers of CpG sites significantly associated with SBP and DBP measures, Figure S2: Gene ontology and KEGG pathways related to 15523 target genes mapped with CpG sites significantly associated with BP measures, Table S1: Hyperparameters of the optimal DL model for each outer CV, Table S2: Comparisons between genes mapped with CpG sites significantly associated with BP and genes regulating high BP in DAVID Bioinformatics Resources 6.8 and DisGeNet databases, Table S3: Estimated associations between significant CpG sites and BP measures.
